# Investigating the Biological Effects of Plant Essential Oils on Plant-Decaying Pathogens

**DOI:** 10.3390/plants15040542

**Published:** 2026-02-09

**Authors:** Hazem S. Elshafie, Aniello Crescenzi, Ippolito Camele

**Affiliations:** Department of Agricultural, Forestry, Food and Environmental Sciences (DAFE), University of Basilicata, 85100 Potenza, Italy; hazem.elshafie@unibas.it (H.S.E.); aniello.crescenzi@unibas.it (A.C.)

**Keywords:** essential oils, biological activity, biochemical characterization, plant diseases, chemical pesticides, pathogenicity, synergistic effect

## Abstract

Essential oils (EOs), complex volatile compounds synthesized by plants, represent a vital class of natural products that are increasingly significant in scientific research due to their diverse biological properties and broad-spectrum medicinal applications. This study provides a comprehensive overview of EOs, commencing with a historical perspective and detailing their applications. It systematically catalogs their primary botanical sources, with specific examples of the most common and important plant families, including *Lamiaceae* (e.g., sage, oregano, thyme), *Verbenaceae* (vervain), *Magnoliaceae* (magnolia), *Rutaceae* (lemon), *Myrtaceae* (eucalyptus) and *Lauraceae* (cinnamon). A key focus is their antifungal activity, including the bioactive constituents involved and their mechanisms of action, with particular emphasis on their defense against pathogenic postharvest fungi. This includes an analysis of the key bioactive constituents responsible for these bioeffects and an exploration of their possible mechanisms of action against phytopathogenic fungi, with particular emphasis on postharvest pathogens infecting several crops. The discussion further highlights the role of EOs as sustainable alternatives to synthetic fungicides for controlling plant diseases that avoid the negative ecological and public health impacts associated with conventional agrochemicals. The study addresses these objectives by describing methods for testing antimicrobial efficacy, including kill-time studies, LD_50_ determination, growth-curve analysis, the poisoned food technique, Spore-germination assays, and metabolic CO_2_ measurement. The current review also highlights some recent studies reviewing the in vitro and in vivo antifungal performance of specific EOs against postharvest diseases.

## 1. Introduction

Essential oils (EOs) are concentrated, water-repelling liquids made up of volatile, aromatic molecules produced by plants. They are sometimes called volatile or ethereal oils. The term ‘essential’ refers to the oil’s characteristic plant aroma, not to any medical or culinary significance.

There is no doubt that EOs have important pharmacological properties, which result in their widespread use in pharmaceutical practices. Buchholtz [1] first noted the antimicrobial potential of EOs, showing that thymol inhibited bacterial growth in tobacco extract more effectively than phenol, the standard surgical antiseptic at the time [2]. Since then, extensive studies have been undertaken to evaluate the antimicrobial potential of numerous EOs already recognized within the pharmaceutical field.

EOs’ fungitoxicity is often due to interactions among their constituents, which act more effectively together than alone [3]. Their effectiveness in vapor form has also been highlighted, making EOs promising candidates for protection of stored food [3,4,5,6].

Unlike broader reviews, this review provides a focused analysis of plant EOs’ effects on postharvest and plant-decaying phytopathogenic fungi. This work emphasizes economically important pathogens responsible for crop losses during both pre- and postharvest stages, with particular attention paid to fungi infecting fruits and vegetables. This review links bioactive EOs and their plant sources with current insights into antifungal mechanisms, including membrane disruption, mitochondrial impairment, oxidative stress, spore inhibition, and synergistic effects. Furthermore, the review highlights recent advances in EO-formulations and delivery strategies, such as nanoemulsions and encapsulation systems, and discusses their practical relevance in in vivo, semi-field, and open-field applications. By combining pathogen-oriented analysis, mechanistic understanding, and applied perspectives, this review aims to bridge the gap between laboratory research and sustainable disease management in agriculture.

The present review is organized into a series of thematically structured sections to provide a comprehensive overview of plant EOs and their biological activity. It opens with a general introduction to plant EOs, with particular emphasis on their diverse biological properties. This is followed by an analysis of conventional chemical strategies for controlling plant pathogenic fungi, including commonly used pesticides and their associated ecological and health-related drawbacks. The subsequent section focuses on plant EOs, detailing the principal botanical families that produce them, including *Lamiaceae*, *Verbenaceae*, *Magnoliaceae*, *Rutaceae*, *Myrtaceae*, and *Lauraceae*. A dedicated section addresses the antimicrobial activity of EOs, with a discussion of their antifungal mechanisms and potential synergistic interactions, including some relevant studies illustrating the practical applications of these oils.

## 2. Conventional Chemical Control of Plant Pathogenic Fungi

Fungi and fungus-like organisms cause more plant diseases than any other pathogen group, with over 8000 infectious species identified. Their roles in plant and human illness, their importance in biotechnology and pharmaceuticals, and their function as decomposers have gained extensive global research attention. Several historical famines and periods of severe human hardship have been linked to destructive fungal pathogens of major crops [7].

Traditional chemical traditional strategies typically depend on compounds that are toxic to target pathogens and operate through specific modes of action, such as commercial fungicides and antibiotics. In an ideal scenario, these chemicals should provide effective control while remaining safe for non-target species and causing minimal disruption to the environment, beneficial microorganisms, and soil ecosystems.

Synthetic fungicides have long been the primary tool for suppressing fungal diseases. Growing awareness of the health and environmental risks of pesticides, along with the loss of key active ingredients, has spurred the search for safer natural alternatives for postharvest disease control [8]. To reduce the likelihood of resistance developing from repeated use of the same chemical class, it has been recommended to rotate fungicides with different modes of action. From the 1980s onward, concerns escalated regarding the hazards posed by agricultural pesticides due to their potential toxicity to humans, beneficial soil organisms, and wildlife, as well as contamination of food crops. These risks arise from dermal absorption, inhalation, or ingestion of pesticide residues. Table 1 outlines the major types of chemical pesticides, as well as their principal plant disease targets, mechanisms of action, and potential adverse effects on human health and the environment.

Mitigating the dangers associated with pesticide use requires careful handling and improved agricultural practices. Environmental concerns have focused on sulfur- and copper-based fungicides, which can harm many organisms when washed into soil or aquatic ecosystems. Many countries have launched farmer training programs aimed at reducing excessive pesticide application and enhancing the production of key crops.

In recent years, there has been growing interest in plant-derived extracts as natural alternatives to synthetic pesticides [3,8]. This trend is largely driven by concerns over environmental contamination and the increasing appearance of pesticide-resistant pests, insects, and fungal pathogens.

Overall, plant EO-based strategies differ fundamentally from chemical fungicides. EOs contain diverse bioactive compounds that target multiple sites in pathogens, such as membranes, enzymes, and signaling pathways. In contrast, chemical fungicides usually act on a single, specific target. This multi-target action of EOs lowers the risk of resistance, whereas pathogens can more easily develop resistance to single-target fungicides. From a sustainability perspective, EOs come from renewable plant sources and are generally biodegradable and pose low environmental and health risks. Although their effectiveness can vary with formulations and conditions, EO-based approaches conceptually support integrated pest management and sustainable agriculture better than conventional chemicals.

## 3. Plant Essential Oils

EOs are aromatic substances synthesized by many plant species. They are believed to contribute to the plant’s natural defense system by providing protection against pathogenic microbes [29,30,31,32,33]. Over time, these oils have been examined in pharmacological research and subsequently evaluated extensively for their antimicrobial properties.

A variety of laboratory techniques have been employed to assess the antimicrobial activity of EOs. Commonly used approaches include agar diffusion assays, broth or agar dilution methods, and tests that measure activity in the vapor phase [34].

Additional antimicrobial evaluation methods include the following:

1. Phenol coefficient (kill-time) assays:

These tests compare the effectiveness of a compound to that of phenol after a 15 min exposure period.

2. Determination of lethal exposure time:

This method measures how long a test substance must be in contact with contaminated silk threads to completely eliminate the target organism.

3. LD_50_ measurement and growth-curve analysis:

Growth curves are generated to identify the concentration of a compound that suppresses growth in 50% of the microbial population.

4. Poisoned food method:

Here, the delay or reduction in microbial growth is assessed when the organism is cultured on media containing inhibitory substances.

5. Spore-germination tests:

Commonly used for fungi, this test evaluates inhibition during early germination, particularly in short-exposure scenarios.

6. Metabolic CO_2_ output monitoring:

This technique detects microbial activity—such as yeast growth—by tracking CO_2_ production or by using color-changing indicators like sulfur salts in supplemented milk or tetrazolium dyes (e.g., TTC or INT).

## 4. Common Bioactive EOs Used as Natural Defenders Against Plant Diseases

### 4.1. Family Lamiaceae

#### 4.1.1. Sage

Sage is a prominent genus within the *Lamiaceae* family, which includes around 900 species distributed across temperate, subtropical, and tropical regions worldwide, with notable concentrations in the Mediterranean, Central Asia, Central and South America, and southern Africa. Among these, *Salvia officinalis* L., *S. fruticosa* Mill., and *S. divinorum* Epling & Játiva, are the most recognized species, widely used in both traditional and modern medicine.

Sage EO exhibits significant antimicrobial activity against several key plant pathogenic fungi responsible for postharvest and field decay. Studies have demonstrated its potent inhibitory effects on mycelial growth and spore germination of fungi such as *Botrytis cinerea* (gray mold), *Aspergillus niger* (black mold), and *Fusarium oxysporum* (vascular wilt). Its antifungal properties are primarily attributed to its high content of bioactive monoterpenes, including 1,8-cineole, camphor, and α-thujone, which disrupt microbial cell membranes and metabolic processes. These findings highlight the potential of sage oil as a natural biocontrol agent for managing plant diseases and reducing reliance on synthetic fungicides [35,36].

#### 4.1.2. Oregano

Oregano is often referred to as wild marjoram, and it is closely related to *Origanum majorana* L., commonly known as sweet marjoram. Over centuries, numerous varieties and strains have been selected for their distinctive aromas and other desirable traits. The leaves are widely used as a culinary ingredient and are often more aromatic when dried than when fresh. Oregano is known for its warm, slightly bitter flavor, though its intensity can differ across cultivars.

Pavarino et al. [37] documented the antifungal and antibacterial effects of oregano EO on a broad range of plant pathogens. These include *Penicillium purpurogenum*, *Staphylococcus aureus* and *Salmonella enterica.* Its primary fungicidal action is attributed to the high concentration of phenolic compounds, especially carvacrol and thymol, which compromise the integrity and function of microbial cell membranes [38].

#### 4.1.3. Thyme

Thyme is commonly represented by *Thymus vulgaris* L. It belongs to the genus *Thymus* within the *Lamiaceae* family and shares close botanical ties with oregano (*Origanum* spp.).

*T. capitatus* (L.) Cav. EO exhibited antifungal properties in stored food products, inhibiting the growth of *Botrytis cinerea* and *Monilinia fructicola* [39]. *T. vulgaris* EO has also demonstrated activity against several postharvest fungal pathogens, including *B. cinerea*, *Penicillium italicum*, *P. citrophthora*, and *Rhizopus stolonifer* [30]. The dominant bioactive components, thymol and carvacrol, are responsible for severe membrane damage, leading to cellular leakage and death in fungal cells. Thyme EO is considered a suitable candidate for controlling both field and postharvest diseases [40].

#### 4.1.4. Marjoram

In a study on *Marjorana hortensis* L. (*Lamiaceae*), known commonly as marjoram, Camele et al. [30] reported that *Marjorana hortensis* EO exhibited antifungal activity against *P. citrophthora* and *R. stolonifera*. *M. hortensis* EO also suppressed *Colletotrichum acutatum* and *B. cinerea*. Its efficacy is largely attributed to its complex chemical profile, where oxygenated monoterpenes like α-pinene, limonene, linalool and terpinene-4-ol act synergistically to disrupt fungal membranes and cellular processes [41]. These findings support the potential of marjoram EO as plant-based alternative for disease management [42].

### 4.2. Family Verbenaceae

#### Vervain

*Verbena officinalis* L., or vervain, has a long history in folk medicine and has recently attracted attention for its potential anticancer properties. Despite its broad use, the underlying mechanisms responsible for its pharmacological activity remain poorly understood.

Elshafie et al. [32] recently demonstrated the antimicrobial effectiveness of thyme and vervain EOs, showing that it significantly reduced brown rot lesions in peaches caused by *Monilinia laxa*, *M. fructicola*, and *M. fructigena* under in vivo conditions.

Other research, including Camele et al. [30], has indicated the inhibitory action of vervain EO against significant agents of crop decay, such as *Fusarium graminearum*, *Aspergillus ochraceus*, and *Alternaria alternata*. Its antifungal mechanism is linked to its major volatile constituents, including citral, geraniol, and limonene, which interfere with fungal cellular integrity and enzymatic activity [43].

### 4.3. Family Magnoliaceae

#### Magnolia

The genus *Magnolia* comprises roughly 210 species of flowering plants within the Magnoliaceae family. These ancient trees have a long history of traditional use, with both their flowers and their EOs valued across many cultures.

EO extracted from *M. liliflora* Desr. has demonstrated strong antifungal activity in vitro and in vivo against a range of plant pathogens, including *B. cinerea*, *Colletotrichum capisci*, *Fusarium oxysporum*, *F. solani*, *Phytophthora capisci*, *Rhizoctonia solani*, and *Sclerotinia sclerotiorum* [44]. Its effectiveness against *P. capsici* in vivo has been attributed to several key constituents, such as β-caryophyllene, α-terpineol, α-humulene, farnesene, β-pinene, and caryophyllene oxide [44].

### 4.4. Family Rutaceae

#### Lemon (*Citrus limon*)

The *Rutaceae* family, or citrus family, is a major source of EO due to oil glands in its fruit peels, leaves, and flowers.

*Citrus limon* (L.) Osbeck (lemon) EO, mainly obtained by cold-pressing the peel, is rich in monoterpenes, especially limonene (up to 70%), with β-pinene, γ-terpinene, and citral [45]. The oil exhibits antioxidant, antimicrobial, and anti-inflammatory activity, making it valuable in pharmaceuticals, cosmetics, and food [46]. Its composition varies with cultivar, origin, and extraction method, emphasizing the need for standardization [45]. Limonene contributes to aroma, and also has cytoprotective and chemopreventive effects, supporting its use in aromatherapy and natural preservatives [47].

### 4.5. Family Myrtaceae

#### Eucalyptus

Eucalyptus (*Eucalyptus* spp. L’Hér.) EO is widely recognized for its potent and antimicrobial properties against some plant pathogenic fungi [48]. Studies have reported that its strong inhibitory effects against major decay agents are attributed to its high concentration of 1,8-cineole (eucalyptol), which damages fungal membranes and disrupts cellular respiration [49].

### 4.6. Family Lauraceae

#### Cinnamon (*Cinnamomum* spp.)

Cinnamon (*Cinnamomum* spp. Schaeff.) EO is one of the most important natural antimicrobial agents, exhibiting potent fungicidal activity against several plant-decaying pathogens, especially against postharvest and soil-borne fungi, including *Botrytis cinerea*, *Rhizoctonia solani*, and *Penicillium expansum* (blue mold). The dominant bioactive component, cinnamaldehyde, is primarily responsible for this strong inhibitory effect, which can damage the fungal cell wall and membrane, leading to cellular leakage and death [50,51].

## 5. Antimicrobial Activity of Plant EOs

Plant-derived EOs have gained significant attention for their broad antimicrobial potential, notably their antibacterial, antifungal and antiviral activity. For instance, a study screening 21 different plant EOs found that 19 exhibited inhibitory activity against at least one of six bacterial species, including Gram-positive *Staphylococcus aureus* and Gram-negative *Escherichia coli*, with oils such as cinnamon, clove, lime, geranium and rosemary being the most effective [52]. Further work demonstrated strong antibacterial performance of oregano, thyme, clove-bud, garlic and other EOs: in one screening across 31 oils, the most potent against *S. aureus* were oregano, garlic, pelargonium, cinnamon and thyme, and against *E. coli*, the ranking was oregano, thyme, wild thyme, clove-bud and peppermint [53]. Mechanistic studies suggest that these oils act primarily via disruption of microbial plasma membranes, leakage of ions and cellular contents, and inhibition of respiration and enzyme systems [54,55,56,57,58].

Beyond bacteria, plant EOs show promising antifungal and antiviral activity. Reviews note that EOs such as those from fennel, coriander, anise, geranium, Japanese mint, cinnamon, clove and lemongrass exhibit strong antifungal effects against various yeasts and filamentous fungi (e.g., *Candida albicans*), even at low concentrations [59]. On the antiviral front, oils from certain *Eucalyptus* species demonstrated activity in vitro against human pathogens (e.g., Herpes simplex virus 1, Coxsackie B4, and Hepatitis A virus), with IC_50_ values around 2–6 µg/mL [60]. These findings indicate that plant EOs are a rich source of antimicrobial agents with multi-kingdom activity (bacteria, fungi, viruses) and could serve as key ingredients for novel anti-infective products.

A wide range of EOs have demonstrated strong antifungal activity against major postharvest pathogens, including *Botrytis cinerea* [61,62,63], species of *Aspergillus* [64,65,66], *Fusarium* spp. [67,68], *Penicillium* spp. [69], *Rhizopus stolonifer* [70,71], and *Colletotrichum gloeosporioides* [72]. Their effectiveness is largely attributed to the suppression of mycelial development as well as the prevention of spore germination. This mode of action implies that disease progression can be limited by hindering both the initial infection process and the subsequent expansion of fungal mycelia beyond the primary infection point [73].

Clove EO, derived from *Syzygium aromaticum* (L.) Merr. & Perry, has demonstrated notable bioactivity, largely due to its major monoterpene, eugenol [74]. This EO has been shown to inhibit the growth of several postharvest pathogens on apples, including *Botrytis cinerea*, *Monilinia fructigena*, *Penicillium expansum*, and *Phlyctema vagabunda*. Similarly, carvacrol, a phenolic compound abundant in oregano (*O. vulgare*) EO, has been reported to strongly suppress mycelial growth of *Neofabraea alba* on apple fruits [75].

Other Mediterranean EOs, such as those obtained from basil (*Ocimum basilicum* L.), fennel (*Foeniculum vulgare* Mill.), lavender (*Lavandula officinalis* Mill.), marjoram (*O. majorana* L.), peppermint (*Mentha piperita* L.), rosemary (*Rosmarinus officinalis* Spenn.), sage (*Salvia officinalis* L.), savory (*Satureja montana* L.), thyme (*T. vulgaris* L.), oregano, and wild mint (*Mentha arvensis* L.), have also shown considerable antifungal potential, in some cases outperforming conventional chemical treatments, particularly against *B. cinerea* and *P. expansum* during postharvest storage of apples [3].

The observed differences in EO effectiveness are primarily attributable to the individual antifungal properties of their chemical constituents, as well as to synergistic interactions among these compounds [3,75]. Some studies suggest that potential phytotoxic effects of EO treatments may arise from the same active molecules responsible for antifungal activity. Additionally, the duration of storage can influence EO efficacy, indicating that EO-based treatments may be most effective over short storage periods or may require repeated applications tailored to the specific fruit cultivar [3]. Selection of an appropriate EO for postharvest use should therefore consider fruit type, storage duration, and the intended control of decay.

Application methods also play a key role in maximizing antifungal performance. Both dipping and spraying techniques have been investigated for delivering EOs onto fruits and vegetables, demonstrating improvements in postharvest disease management [76,77,78]. Combining EO treatments with other postharvest strategies has been shown to enhance overall efficacy against spoilage organisms [73,79,80].

Specific EOs, including thyme (*T. capitatus*), spearmint (*M. spicata* L.), and anise (*Pimpinella anisum* L.), have exhibited inhibition control effects on *M. fructicola* development [65,66]. Lemon myrtle (*Backhousia citriodora* F.Muell.) EO has also displayed strong antifungal activity against this pathogen, largely due to its major constituent, citral, which has been identified as a potent fungitoxic agent with potential applications in topical pharmaceuticals and postharvest disease control [39].

Although the antimicrobial activity of many plant EOs is based on controlled in vitro or greenhouse experiments, the direct quantitative comparison of application doses with synthetic fungicides is not the real target at present. These chemical products are highly optimized formulations designed for field use and are effective at low concentrations but have negative impacts on non-target organisms as well as soil and human health, and lead to pathogen resistance development. When synthetic fungicides are prohibited, EOs represent not only alternatives but leading candidates for sustainable crop protection, supporting the reduction in chemical residues, protection of beneficial organisms, and consumer demand for healthy food.

## 6. Antimicrobial Action of EO-Constituents Against Plant Pathogens

The potent antimicrobial properties of plant EOs against phytopathogens are primarily attributed to a diverse array of secondary metabolites, predominantly belonging to the terpenoid and phenylpropanoid chemical families [39]. These bioactive constituents, which often act in a synergistic way within the whole oil, target fundamental structures and processes in fungal and bacterial cells, leading to growth inhibition, Spore-germination failure, and cell death.

The most common bioactive constituents can be categorized into the following based on their chemical class and primary mode of:

### 6.1. Phenolic Compounds

Phenolic compounds such as carvacrol and thymol (abundant in oregano and thyme oils) [31,33], and the aldehyde cinnamaldehyde (from cinnamon oil) [38], are among the most powerful compounds. Their antimicrobial action is largely non-specific and concentration-dependent. Their hydroxyl (-OH) or aldehyde (-CHO) groups impart a strong lipophilic character, enabling them to integrate into and disrupt the microbial plasma membrane. This integration increases membrane permeability, leading to the leakage of vital cellular ions (e.g., K^+^, H^+^) and ATP, collapse of the proton motive force, and ultimately, cytolysis. 

### 6.2. Monoterpene Hydrocarbons

Monoterpene hydrocarbons like limonene and pinenes contribute to membrane fluidity and disruption, but are generally less potent than their oxygenated derivatives [41]. In contrast, oxygenated monoterpenes, including alcohols (e.g., linalool, terpinen-4-ol in marjoram), aldehydes (e.g., citral in vervain), and ketones (e.g., camphor in sage), exhibit stronger activity. Their mechanism extends beyond membrane disruption to include the inhibition of key enzymes, interference with energy metabolism (e.g., respiration), and the disturbance of ion channel function. A particularly widespread and significant oxygenated monoterpene is the ether 1,8-cineole (eucalyptol), the major component of many eucalyptus oils. It is known to cause extensive membrane damage and is also a potent inhibitor of fungal sporulation and mycotoxin production.

The specific action of these compounds causes a series of problems inside plant pathogens. In fungi, the first effect is damage to the cell membrane, which slows or stops hyphal growth and germ tube development. Importantly, many EOs and their active compounds strongly reduce or completely block spore germination, which is a critical step for the start and spread of plant diseases. Furthermore, certain components, like thymol and cinnamaldehyde, can denature proteins and inhibit enzymes critical for cell wall synthesis (e.g., chitin synthase in fungi) or virulence factor production. The efficacy of a given constituent is influenced by its concentration and the presence of other compounds in the oil, which can create synergistic effects.

## 7. Mechanism of Antifungal Activity and Synergistic Interactions of EOs

Plant EOs have been extensively studied for their potent antimicrobial properties, which originate from their complex mixture of bioactive compounds, including phenolic compounds, monoterpenes, sesquiterpenes, alcohols, aldehydes, and other small volatile molecules [81,82,83,84]. These molecules act through multi-target mechanisms, simultaneously affecting multiple cellular structures and pathways within fungal pathogens. EOs target multiple cellular sites, unlike conventional fungicides that act on a single site, reducing the likelihood of resistance.

A central antifungal mechanism of EOs involves disruption of the fungal cell wall and plasma membrane. Lipophilic constituents, such as monoterpenes, phenols, and aldehydes, interact with membrane lipids and embedded proteins, with particular affinity for ergosterol, a sterol that is critical for fungal membrane integrity. This interaction alters membrane fluidity, permeability, and overall stability, leading to the leakage of ions, nucleotides, and proteins from the cytoplasm. The resulting disturbance of cellular homeostasis impairs essential physiological processes, including spore germination, hyphal elongation, and nutrient transport, ultimately causing cell death [54,85].

Beyond membrane-targeted effects, EO components can impair mitochondrial function, uncouple oxidative phosphorylation, and deplete intracellular ATP pools, thereby compromising the energy balance of fungal cells. Simultaneously, many EO constituents induce the accumulation of reactive oxygen species (ROS), which damage proteins, lipids, and nucleic acids, resulting in oxidative stress that further enhances antifungal activity [86]. The simultaneous targeting of multiple pathways—membranes, mitochondria, and oxidative homeostasis—reduces the potential for fungal adaptation or resistance, a common drawback of single-site fungicides.

The antifungal activity of EOs is further enhanced by synergistic interactions among their constituent molecules. Combinations of monoterpenes and phenolic compounds often exhibit inhibitory effects that exceed the additive activity of each individual compound [37]. For example, the monoterpene p-cymene, which on its own has relatively weak antifungal activity, can increase the permeability of the fungal membrane, facilitating the cellular uptake of the phenolic compound carvacrol and thereby amplifying its toxicity. Other phenolic and terpenoid constituents, such as thymol, eugenol, and carvone, act cooperatively, targeting both membrane integrity and enzymatic processes within the fungal cell [33]. In addition, EOs can disrupt cell wall enzymes, leading to fragile, thin, and poorly branched hyphae that limit fungal growth [87,88].

Synergistic effects are also observed when whole EOs are blended. In these mixtures, one oil may primarily disrupt membrane integrity while another interferes with enzymatic activity, mitochondrial respiration, or ROS regulation [89,90]. Notable EO combinations—oregano with thyme, and clove with cinnamon—significantly inhibit human and plant pathogens like *Candida albicans* and *Aspergillus* spp., often cutting individual MICs by over 50% [91]. Such combinations allow for the use of lower concentrations, reducing potential phytotoxicity, volatility, and organoleptic effects, while maintaining strong antifungal efficacy.

The biochemical composition of EOs is a key determinant of their antifungal potency. *Origanum vulgare* ssp. *hirtum* EO, for instance, exhibits a chemotype dominated by the phenolic compounds carvacrol and thymol. This oil demonstrated strong inhibitory activity against *Monilinia* spp. (*M. laxa*, *M. fructigena*, and *M. fructicola*) at concentrations of 250–1000 ppm in vitro on PDA plates containing 0.2% Tween-20, without inducing phytotoxic effects or hemolysis [31,92]. Beyond oregano, Mediterranean aromatic plants such as thyme (*Thymus vulgaris*) and vervain (*Verbena officinalis*) also produce EOs with broad-spectrum antimicrobial properties. These oils have been shown to inhibit multiple bacterial and fungal plant pathogens, including *Aspergillus*, *Fusarium*, *Penicillium*, *Pseudomonas aeruginosa*, *Staphylococcus aureus*, *Clavibacter michiganensis*, *Phytophthora infestans*, and *Sclerotinia sclerotiorum*. In particular, *O. vulgare* and *O. syriacum* display notable activity against *Botrytis cinerea*, confirming their potential in postharvest disease management [30,37,76,77].

The antifungal potential of EOs is not limited to in vitro conditions. In vivo studies have demonstrated that thyme and vervain EOs effectively reduce disease severity in postharvest peach fruits. Thyme EO, characterized by high o-cymene content (56.2%), and vervain EO, rich in citral (44.5%) and isobornyl formate (45.4%), significantly reduced the diameters of lesions caused by *Monilinia* species when applied at 250–500 ppm (thyme) and 500–1000 ppm (vervain), without causing phytotoxic effects [32].

The activity of individual EO constituents has also been extensively evaluated. Studies show that citral exhibits fungicidal activity against *P. citrophthora*, while carvacrol and thymol primarily act as fungistatic agents against *P. citrophthora* and *R. stolonifer*. Thymol additionally inhibits *P. italicum*, and complete inhibition of *B. cinerea* is achieved with citral and carvacrol at 250 ppm and thymol at 150 ppm [93]. Further investigations on *Monilinia* infections in peach fruits confirmed that emulsions of carvacrol and thymol at 150–500 ppm strongly reduce lesion development, demonstrating the practical in vivo antifungal efficacy of these compounds [33].

These findings show that EOs’ broad antifungal activity stems from multiple bioactive molecules targeting membranes, enzymes, mitochondria, and oxidative balance. This multi-target mechanism underlies their effectiveness against major plant pathogens such as *Botrytis cinerea*, *Alternaria* spp., and *Fusarium* spp., while minimizing the risk of resistance development. Although complete mycelial inhibition requires high in vitro EO concentrations (200–500 ppm), modern formulations—nanoencapsulation, emulsions, and EO-based coatings—improve stability, prolong bioactivity, and lower practical doses. By combining chemical profiling, mechanistic understanding, and empirical evaluations of both whole oils and their individual constituents, EOs can be effectively harnessed as sustainable antifungal agents in crop protection.

Recent research under open-field and semi-field conditions (in vivo trials) has increasingly demonstrated the practical efficacy of plant EOs for controlling economically significant plant-decaying pathogens. For instance, in open-field trials against tomato late blight (*Phytophthora infestans*), thyme and cinnamon EOs applied as foliar sprays significantly reduced disease severity, showcasing performance comparable to some synthetic fungicides [88]. Similarly, semi-field studies on strawberry anthracnose (*Colletotrichum* spp.) demonstrated that clove and lemongrass oil formulations effectively suppressed fruit rot, highlighting their potential in high-value-crop protection. These studies show that EOs both inhibit pathogens and induce systemic resistance, providing multi-modal defense even under high natural-pathogen pressure.

To address inherent limitations of EOs—such as volatility, photodegradation, and poor water solubility—advanced formulation strategies are essential for enhancing field performance. Nanoemulsions and encapsulation techniques have shown particular promise. For example, encapsulated thyme or garlic oil nanoemulsions demonstrate improved stability, prolonged leaf persistence, and enhanced efficacy against pathogens like *Botrytis cinerea* in grapevines and *Alternaria solani* in potatoes [94].

These formulations increase practical utility by enabling controlled release, protecting active compounds from degradation, and improving coverage and uptake, effectively translating in vitro activity into reliable disease management under real-world conditions. Although comprehensive economic assessments are limited, evidence suggests that well-formulated, sustainably sourced plant EOs can provide a competitive and environmentally friendly alternative—or complement—to conventional synthetic fungicides.

**Figure 1:**
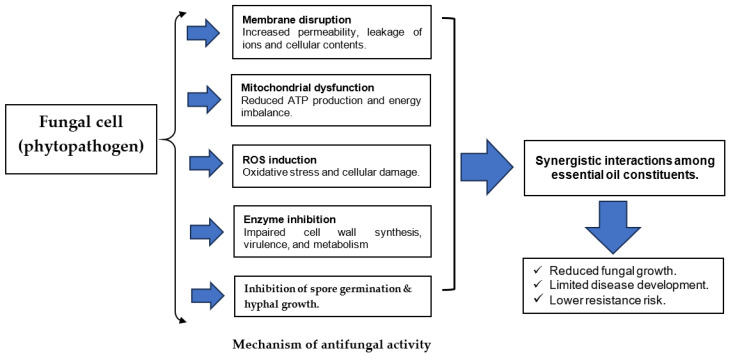
Schematic diagram illustrating the main mechanisms of antifungal action of essential oils.

Overall, the antifungal activity of plant EOs and their bioactive constituents results from a coordinated, multi-target mode of action (Figure 1). Key mechanisms include disruption of fungal cell membrane integrity and permeability, leading to leakage of ions and vital cellular components; impairment of mitochondrial function and energy metabolism; induction of oxidative stress through reactive oxygen species (ROS) accumulation; and inhibition of essential enzymatic processes involved in cell wall synthesis, virulence, spore germination, and hyphal development. These effects often act simultaneously and are frequently enhanced by synergistic interactions among individual constituents or between blended EOs. This combined biochemical action disrupts fungal cell balance, slows pathogen growth, and lowers the risk of resistance, thereby supporting the broad-spectrum effectiveness of EOs against plant-decaying and postharvest pathogens.

## 8. Future Research Perspectives on the Bio-Applications of EOs

Future research on the biological applications of plant EOs should focus on overcoming limitations related to stability, volatility, and delivery while simultaneously enhancing their efficacy and safety in both agricultural and biomedical contexts. Advances in nanoencapsulation, emulsification technologies, and polymer-based coatings offer promising strategies to improve EO persistence, control release rates, and reduce phytotoxicity, enabling more reliable and effective field applications. Integrating chemical profiling with omics technologies—including genomics, transcriptomics, and metabolomics—will help elucidate precise molecular targets, resistance mechanisms, and pathways affected by EO constituents. Further studies should investigate synergistic interactions between EOs, their individual compounds, and conventional antifungal or antibacterial agents, potentially reducing required doses and mitigating resistance development. Expanding in vivo evaluations and field trials remains essential to validate laboratory findings under real environmental conditions, particularly concerning postharvest disease management and crop protection. Comprehensive safety assessments involving toxicology, environmental impact, and non-target effects will also guide regulatory acceptance.

Although comprehensive economic analyses remain limited, existing evidence suggests that plant EOs—especially when locally sourced and appropriately formulated—may represent a competitive, cost-effective, and environmentally sustainable alternative or complement to synthetic fungicides. Their broader adoption has the potential to reduce chemical residues, lower environmental toxicity, and support farm-level circular economies; however, long-term cost–benefit evaluations addressing yield stability, formulation scalability, and production consistency are still required

Finally, systematic exploration of underutilized aromatic plants spices, region-specific chemotypes, and sustainable extraction technologies could expand the diversity and availability of bioactive EO compounds. Collectively, these research directions will facilitate the development of robust, eco-friendly EO-based biopesticides and therapeutic agents with enhanced practical applicability.

## 9. Conclusions

Plant EOs have emerged as promising, eco-friendly alternatives to synthetic pesticides for managing a broad spectrum of phytopathogenic fungi. As evidenced in this review, a wide range of EOs and their formulations, such as oregano, thyme, vervain, magnolia, lemon, eucalyptus and cinnamon, along with their bioactive constituents (e.g., carvacrol, thymol, linalool, and citral), demonstrate potent antifungal activity both in vitro and in vivo. Their mechanisms, which can interfere with essential fungal processes, enable effective inhibition of mycelial growth and spore germination, often at low concentrations. These findings support their incorporation into practical applications such as seed treatments or soil amendments, thereby protecting crops and reducing postharvest losses. However, translating this promising efficacy to open-field conditions requires addressing critical challenges related to formulation, stability, and optimal application dosage. In summary, the novelty of this review lies in its targeted focus on plant-decaying and postharvest pathogens, its integrated discussion of antifungal mechanisms and synergistic interactions of EO constituents, and its emphasis on formulation strategies and field applicability. By linking chemical composition, biological activity, and practical deployment, this work extends beyond descriptive surveys and provides a comprehensive framework for the development of EO-based tools in sustainable crop protection and postharvest disease management. Future research should, therefore, focus on these aspects, particularly for high-impact fungal pathogens, to enable the effective integration of EOs as reliable tools in organic and integrated pest management programs.

## Figures and Tables

**Table 1:** Common chemical pesticides for common plant diseases and their negative impacts.

Chemical Pesticide ^(a)^	Common Target Plant Diseases/Pathogens	Mechanism of Antimicrobial Effect (Mode of Action)	Collateral Negative Impact on Human Health	Collateral Negative Impact on the Environment	References
Chlorothalonil (e.g., Bravo, Daconil, and Echo)	Effective against various fungal plant diseases, including blights, mildews, rusts, and leaf spots.	Multi-site inhibitor interfering with fungal enzymes.	May be associated with respiratory irritation, skin sensitization, endocrine disruption, and potential carcinogenic risks.	Highly toxic to aquatic animals and persistent in soil and water.	[9,10]
Mancozeb (e.g., Penncozeb, Trimanoc, and Vondozeb)	Controls blight, rust, leaf spot, and scabs across a wide range of fruits, vegetables, nuts, and field crops.	Multi-site inhibitor that disrupts multiple enzymes.	Causes several health problems, mainly hepatic, renal and genotoxic.	Moderately persistent, toxic to aquatic organisms, and capable of affecting soil microflora.	[11,12,13,14]
Azoxystrobin (e.g., Amistar, Heritage, and Ortiva)	Rusts, mildews, blights, and fruit rots.	Inhibits mitochondrial respiration.	Exhibits low to moderate toxicity and can induce allergic dermatological reactions.	Toxic to non-target organisms, and persistent in soil, affecting microbes and causing oxidative stress in aquatic life.	[15,16]
Propiconazole (e.g., Tilt, Banner Maxx II, and Patch Pro)	Rusts, powdery mildew, leaf spot, and anthracnose.	Inhibits ergosterol synthesis (DMI fungicide).	May disrupt hormones, cause skin irritation, and affect reproductive health.	Persistent in soil and toxic to aquatic invertebrates and fish.	[17,18]
Copper-based fungicides (e.g., copper hydroxide, copper sulfate, copper oxychloride, copper oxide, and copper octanoate)	Wide range of plant diseases: downy mildew, powdery mildew, blight, leaf spot and bacterial diseases such as fire blight.	Cause cell membrane disruption, produce toxic reactive oxygen species (ROS), and interfere with vital enzymes and nucleic acids.	Causes skin and eye irritation, makes it dangerous to swallow, and might affect the liver or kidneys.	Persists in soil, accumulates in waterways, and toxic to beneficial soil microbes.	[19,20,21]
Sulfur fungicides (e.g., elemental and lime sulfur)	Powdery mildew on fruit trees (fruits), and other broad-spectrum effect against rusts, molds and scabs.	Disrupts fungal respiration.	Hinders lung function and causes respiratory symptoms in children and may also cause skin and eye irritation.	Excessive application acidifies soil, harms beneficial arthropods, and pollutes air.	[22,23]
Captan (e.g., Orthocide, Merpan, and Captan 50 W)	Scabs, blights, brown rot, powdery mildew, and gray mold on fruits, vegetables, and ornamentals.	Multi-site disruptor affecting enzymes and cell membranes.	Leads to skin and eye irritation and is suspected to cause carcinogenicity.	Toxic to aquatic organisms, and breaks down into more persistent metabolites.	[24,25,26]
Carbendazim/Benomyl (e.g., Samartha, Agrocit, and Benex)	Controls powdery mildew, leaf spot, anthracnose, scabs, and Fusarium wilt.	Inhibits fungal cell division.	Has potential teratogenic effects and causes reproductive toxicity.	Persistent, and toxic to earthworms and aquatic life, with rapid resistance development.	[27,28]

^(a)^ The names between brackets correspond to the trademarks of active ingredient.

## Data Availability

The original contributions presented in this study are included in the article. Further inquiries can be directed to the corresponding author.
